# Editorial: Translating clinical genomics and health informatics into precision oncology

**DOI:** 10.3389/fgene.2022.1029212

**Published:** 2022-10-03

**Authors:** Hsih-Te Yang, Dana C. Crawford, Mohamed E. Abazeed

**Affiliations:** ^1^ Atrium Healthcare, Charlotte, NC, United States; ^2^ Case Western Reserve University, Cleveland, OH, United States; ^3^ Feinberg School of Medicine, Northwestern University, Chicago, IL, United States; ^4^ Robert H. Lurie Comprehensive Cancer Center, Northwestern University, Chicago, IL, United States

**Keywords:** precision oncology, personalized medicine, clinomics, panomics, clinical prediction models

Some cancers are driven by targetable genomic alterations that dysregulate key pathways influencing cell growth and survival. These tumors lend themselves to significant, although inevitably transient, clinical responses upon targeted drug treatment. Accordingly, omic-based theranostics (*i.e*. diagnostics that guide therapeutic interventions) have transformed the management of a substantial number of cancers (King et al., 1985; Druker et al., 2001; Slamon et al., 2001; Demetri et al., 2002; Lynch et al., 2004). However, after a highly productive discovery phase resulting in the identification of multiple clinically actionable genomic targets, new information capabilities have been limited by challenges in target druggability, treatment toxicity, and intratumoral heterogeneity. Therefore, the ability to harness tumor omic information to its full clinical potential has not yet been realized.

The impact of these challenges has led to a pivot toward a more comprehensive understanding of the complexity of tumor information and the role of distinct biological processes in driving tumor phenotypes and, hence, patient outcomes. To achieve the requisite level of (epi)genome-phenome knowledge, the interrogation of large-scale datasets for integral biological pathways that regulate tumor behavior has come into greater focus. Technological developments in high-content biological and data platforms have led to the generation of large-scale multi-omics datasets in radiomics, genomics, epigenomics, transcriptomics, proteomics, metabolomics, and phenomics (Song et al., 2020). Collections of publicly available datasets like The Cancer Genome Atlas (TCGA) and The Cancer Imaging Atlas (TCIA) provide critical resources to a research environment that is frequently hindered by access to clinically relevant big data (TCGA Research Network; Cancer Imaging Program). These and similar datasets can have broad utility in advancing a more comprehensive and integrated understanding of indivdiual cancers.

The contributions within this Research Topic are in line with ongoing efforts to better characterize the associations between omic determinants and tumor phenotypes. 1) Li et al. explored a putative role for circular RNA (circRNA) in predicting the immune landscape of lung adenocarcinoma. Their data suggests that a circRNA-related risk score model is associated with the level of immune cell infiltration in the tumor that could impact the efficacy of adjuvant treatments (chemotherapy, immunotherapy, or targeted agents). 2) Chen et al. studied the impact of germline copy number variants (gCNV) on the prognosis of patients with non-small cell lung cancer (NSCLC). They developed a prognostic nomogram model and validated their findings in an external cohort. Whether the two gCNVs they have identified, CNVR395.1 and CNVR2239.1, regulate or are accidentally associated with (*i.e.,* represent a confounder) NSCLC phenotypes require experimental validation. 3) Ye et al. examined the prognostic utility of a signature of ferroptosis, a form of programmed oxidation-related cell death associated with iron accumulation, a decrease in antioxidant cellular capacity, and the accumulation of lipid peroxidation or reactive oxygen species (ROS). In addition to clinical and functional studies validating aspects of this signature, a nomogram that incorporated clinical features provided reasonable concordance with overall survival. 4) Lastly, Wang et al. studied the association of long non-coding RNAs (lncRNA) with prognostication in NSCLC. They reveal lncRNA that are associated several clinical and pathological variates including histology, tumor size, stage, and, relatedly, overall survival. Altogether, these studies represent varied attempts to link omic variates, biological pathways, and patient outcomes. Many more attempts that integrate the panoply of omic variates from bulk and single cells coupled with well annotated clinical outcomes are needed.

The convergence of technology and large-scale dataset development has created an unparalleled opportunity to couple omic information with clinical outcomes for many patients with cancer. Rigorous evaluations will require many additional validations and innovations that are guided by additional advances in tumor omic profiling, data interpretation and integration, and innovative clinical trial designs. The rigor with which these studies are validated and prospectively tested will determine whether and how comprehensive tumor information is incorporated into the routine care of patients. Despite the stated potential, there remain several pitfalls for the informed incorporation of omic data for clinical testing and use. A main hurdle is the risk that large-scale association data emerges without evidence-based clinical approaches for data interpretation and integration. It will be pivotal to proactively construct scientific frameworks with adequate guardrails that can prioritize tumor omic information for clinical use.
